# Superdiffusion-like behavior in zero-temperature coarsening of the $d=3$ Ising model

**DOI:** 10.1038/s41598-023-39328-7

**Published:** 2023-08-15

**Authors:** Denis Gessert, Henrik Christiansen, Wolfhard Janke

**Affiliations:** 1 Institut für Theoretische Physik, Universität Leipzig, IPF 231101, 04081 Leipzig, Germany; 2 Centre for Fluid and Complex Systems, Coventry University, Coventry, CV1 5FB UK; 3 Present Address: NEC Laboratories Europe GmbH, Kurfürsten-Anlage 36, 69115 Heidelberg, Germany

**Keywords:** Ferromagnetism, Phase transitions and critical phenomena, Phase transitions and critical phenomena, Statistical physics, Computational science

## Abstract

One key aspect of coarsening following a quench below the critical temperature is domain growth. For the non-conserved Ising model a power-law growth of domains of like spins with exponent $\alpha = 1/2$ is predicted. Including recent work, it was not possible to clearly observe this growth law in the special case of a zero-temperature quench in the three-dimensional model. Instead a slower growth with $\alpha <1/2$ was reported. We attempt to clarify this discrepancy by running large-scale Monte Carlo simulations on simple-cubic lattices with linear lattice sizes up to $L=2048$ employing an efficient GPU implementation. Indeed, at late times we measure domain sizes compatible with the expected growth law—but surprisingly, at still later times domains even grow superdiffusively, i.e., with $\alpha > 1/2$. We argue that this new problem is possibly caused by sponge-like structures emerging at early times.

## Introduction

To quantify the kinetics of coarsening processes, i.e., their time evolution from a disordered to the preferred equilibrium state at low temperature, is of major interest in many physical systems^1,2^. The studied systems have become more and more complex over the last decades ranging from investigations of interface growth^3,4^ over systems with long-range interactions^5,6^ to the application of the methods to the study of the collapse dynamics of polymers^7,8^. Of technical relevance is this process for example in the fabrication of glasses^9^.

The theory based on deterministic continuum models, predicts for *d*-dimensional systems with short-range interactions and non-conserved *O*(*n*) models for all quench temperatures *T* below the critical temperature $T_c$ a power-law growth of the characteristic length scale of the coarsening domain patterns,

1 $$\begin{aligned} \ell (t) \sim t^{\alpha } \end{aligned}$$

with $\alpha =1/2$ for all systems with ${d>n}$ or ${n>2}$ (Ref.^1^). This was confirmed in numerous simulation studies of quenches to $T\ne 0<T_c$ for such models^1,2,10^. Also in experiments of the ordering kinetics in Cu_3_Au^11^ and at the isotropic-to-cholesteric liquid crystal transition^12^, a value close to $\alpha =1/2$ was reported. For the special case of a quench to $T=0$ in the $d=2$ Ising model a power law with growth exponent $\alpha =1/2$ is observed as well^13,14^.

Somewhat as a surprise, for a long time, numerical simulations of the coarsening in the $d=3$ Ising model when quenched to zero temperature only reported anomalously small values of $\alpha < 1/2$ (Ref.^15^), even though many numerical studies were conducted studying the properties of this system^13,14,16–20^. Often reported are values of $\alpha \approx 1/3$ (Ref.^13,14,16^) when using system sizes of up to $L=240$. This lower exponent has been attempted to be explained in various ways. One such attempt targeted on finding arguments and physical explanations for this phenomenon through the fact that the initial $T=\infty$ structure does percolate in three dimensions but not in two dimensions^14^.

**Figure 1:**
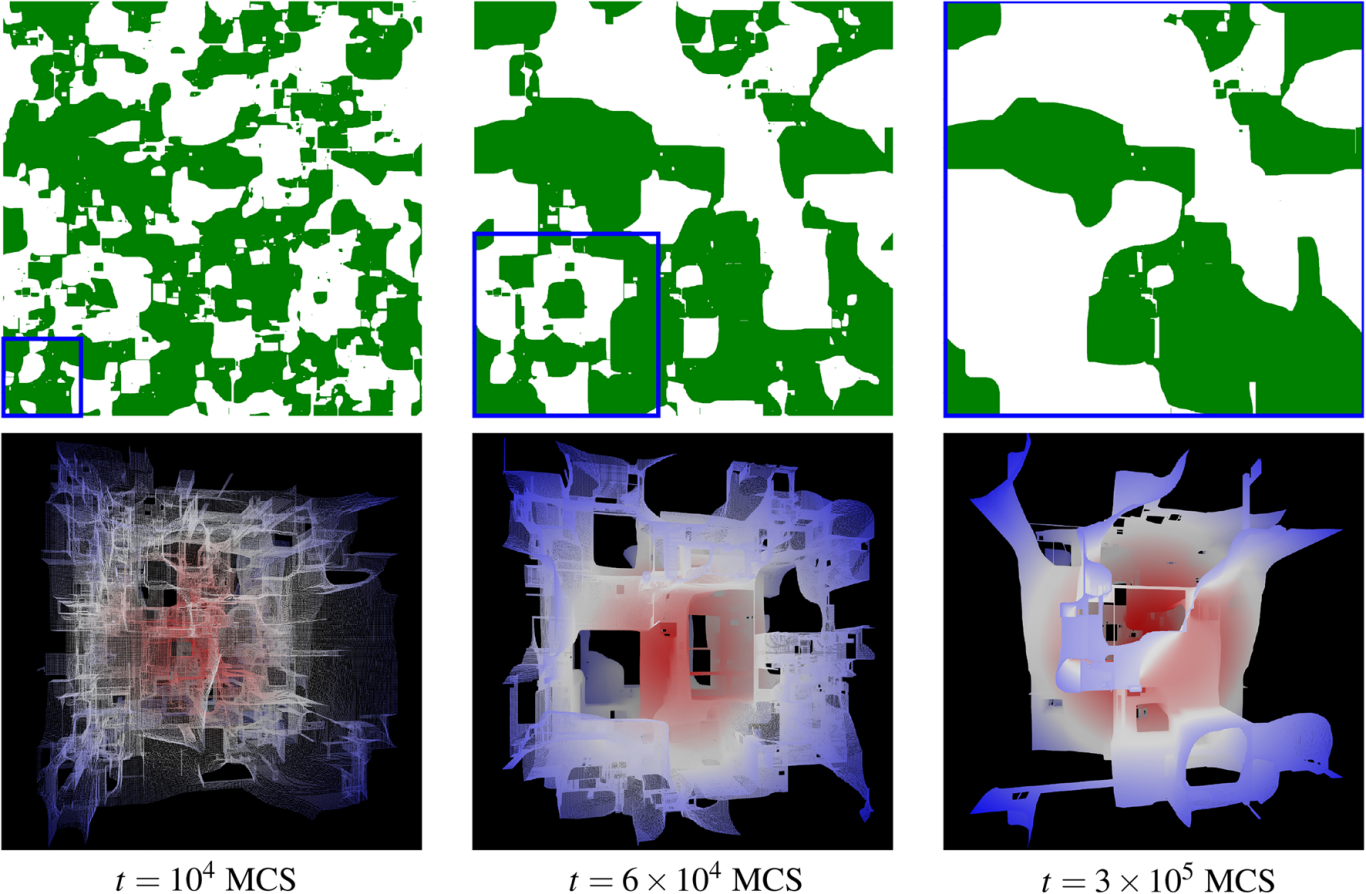
Visualization of the three-dimensional Ising configurations at different times of the quench. Cross-sections (top) and three-dimensional snapshots (bottom) of the spin configuration for $L=2048$ at various times in units of Monte Carlo sweeps (MCS). The cross-sections in the top panel are cuts through the full lattice, where down-spins (the minority direction in this realization) are colored green. The marked square subsections of linear extension $K \simeq 10\,\ell (t)$ (blue boxes) are shown in the bottom panels as three-dimensional visualizations highlighting the interfaces between domains. The color (red-white-blue) indicates the distance from the center of the subsection. For details on the visualizations see Supplementary Section III and for snapshots at more times see Supplementary Fig. 5.

Nonetheless, direct simulations of the continuous and deterministic time-dependent Ginzburg-Landau equation for big systems have provided the correct value of $\alpha =1/2$ (Ref.^21^). In recent work^22–24^ the three-dimensional problem was tackled again by simulating this process using very big simple-cubic lattices with linear size up to $L=750$, from which the authors conjectured a crossover to $\alpha =1/2$ at late times.

**Figure 2:**
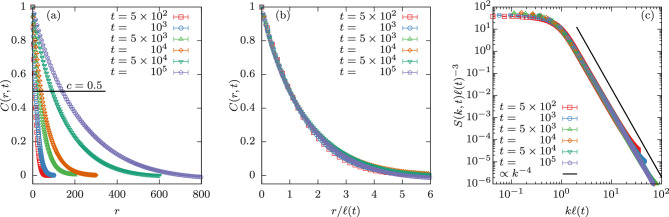
Demonstration of scaling of the two-point correlation function and structure factor. (**a**) Correlation function *C*(*r*, *t*) versus distance *r* for $L=2048$ and several times $t=500,\dots ,10^5$. For increasingly later times, the correlation function decays slower, indicative of a growing length scale. (**b**) Showcase of self-similarity by plotting *C*(*r*, *t*) against $r/\ell (t)$. (**c**) Structure factor *S*(*k*, *t*) scaled to collapse, i.e., $S(k,t)\ell (t)^{-3}$ against $k\ell (t)$. The solid line is a power law $\sim k^{-4}$, where $-4$ is the expected exponent of Porod’s law. Error bars correspond to the standard error.

In an attempt to solve this long-standing puzzle, we performed Monte Carlo (MC) simulations of much larger systems with up to $L=2048$ (using periodic boundary conditions), corresponding to more than 8 billion spins by employing a memory and time efficient GPU implementation. Our implementation is adapted from a publicly available code^25^ that uses a checkerboard decomposition of the system.

## Results and discussion

In Fig. 1 we present visualizations of the lattice configuration of an exemplary simulation run for $L=2048$ at times $t=10^4$, $6\times 10^4$, and $3 \times 10^5$ in units of MC sweeps (MCS). The top row shows plane cuts of the configuration allowing for an easy comparison with the well-known smooth behavior in $d=2$ (see, e.g., Fig. 2 in Ref. ^1^). Three-dimensional representations of domain interfaces are shown in the bottom panel. For early times (left panels), one observes a roughening of the domain boundaries as reported several times earlier for zero-temperature quenches in $d=3$ spatial dimensions. This is clearly in violation of the arguments used to derive $\alpha =1/2$ where a diffusive domain-curvature minimization is assumed, so that here another effective growth exponent is to be expected. Contrasting, at intermediate and even more so at late times (middle and right panels) the domains appear much smoother and diffusion-like growth might be anticipated. However, as domains inside domains are a prominent feature in earlier snapshots but not as much at late times, during the coarsening process annihilation of these domains has to take place. We conjecture that this annihilation is an additional contribution to the domain growth.

To quantify these observations, we measure the two-point equal-time correlation function

2 $$\begin{aligned} C(r,t) = \langle s_i s_j \rangle - \langle s_i\rangle \langle s_j\rangle , \end{aligned}$$

where $\langle \cdot \rangle$ denotes the average over initial conditions and independent trajectories. With increasing order of the system, one expects the correlation function to decay slower, i.e., for late times the correlation function should correspondingly indicate a stronger correlation. Demonstration of this is shown in Fig. 2a for the times mentioned in the key for $L=2048$ and $T=0$. All data was obtained by starting from random spin configurations (with magnetization $m \approx 0$) and averaging over 40 independent realizations (we use the same number of independent realizations for each system size). Note, that previous work^26^ indicates that similar results are to be expected from finite starting temperatures.

*C*(*r*, *t*) is expected to follow dynamical scaling, i.e.

3 $$\begin{aligned} C(r,t) = {\tilde{C}} \left( r / \ell \left( t\right) \right) . \end{aligned}$$

This is self-consistently tested by extracting $\ell (t)$ from the intersection of *C*(*r*, *t*) with a constant value of $c=0.5$. (For the effect of different choices of *c*, see Supplementary Discussion III.) Subsequently we plot *C*(*r*, *t*) versus $r/\ell (t)$ in Fig. 2b. We note that especially at large distances *r* the data collapse is not optimal. This may be an indication of a number of things, e.g., the occurrence of finite-size effects. Another indicative explanation is that the growth exponent $\alpha$ is not yet a constant but effectively varies with *t*.

Additionally, one looks at the structure factor *S*(*k*, *t*) which is the Fourier transform of the correlation function. This quantity, similarly to the correlation function, collapses when properly rescaled, i.e., by plotting $S(k,t)\ell (t)^{-3}$ versus $k\ell (t)$ as shown in Fig. 2c. The data for different sizes collapses quite well and shows a clear power-law decay with Porod’s exponent $d+1=4$.

**Figure 3:**
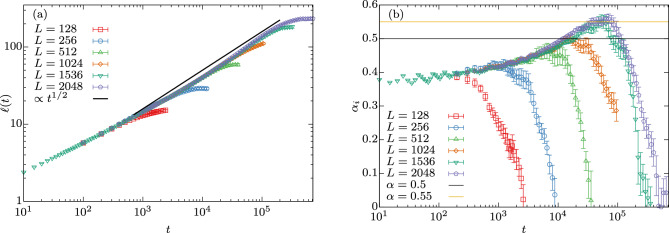
Length scale as a function of time and instantaneous growth exponent. (**a**) Length scale $\ell (t)$ for quenches to $T=0$ in $d=3$ spatial dimensions with linear size $L=128,\dots ,2048$ on a log-log scale. The black solid line shows the expected power-law behavior of $\ell (t) \sim t^{1/2}$. (**b**) Instantaneous exponent $\alpha _i$ is shown against *t* for the same data. Here the *x*-axis is logarithmic to highlight the large *t* behavior where $\alpha _i$ takes values consistently larger than 1/2. Error bars correspond to the standard error.

Finally, we present the characteristic length $\ell (t)$ versus *t* on a log-log scale in Fig. 3a for system sizes $L=128$, 256, 512, 1024, 1536, 2048. Initially, $\ell (t)$ grows independently of the system size *L* as individual domains can grow unrestrictedly. It is only when $\ell (t)$ reaches a value of the order of magnitude of *L* that domain growth is hindered by the finite nature of the lattice which shows itself in the form of finite-size effects that end in a stagnation of the growth for that system size. The solid line shows the asymptotic growth law $\ell (t) \sim t^{1/2}$ as predicted, where one clearly sees that this is not parallel to the data for late times. To get a more detailed impression of this, we show in Fig. 3b the instantaneous exponent

4 $$\begin{aligned} \alpha _i(t) = \frac{d \ln \ell (t)}{d \ln t}, \end{aligned}$$

that is, the local slopes in Fig. 3a. At very early times $\ell (t)$ grows like $t^{\alpha _i}$ with $\alpha _i$ compatible with 0.35–0.40. When only considering lattice sizes up to $L=256$, then only this behavior can be seen as was the case in Refs. ^13,14,16^. Reference ^27^ observed $t^{0.43}$ using a lattice size of $L=512$, which is in good agreement with our measurement for this size. For $L=1024$ we observe $\alpha _i \approx 1 / 2$ for a short time, and for $L=1536$ and $L=2048$ we observe an exponent $\alpha _i > 1/2$, which is completely unexpected from existing simulations and theory. As the two largest system sizes show the $\alpha _i(t) > 1/2$ signal before the onset of finite-size effects in either system size, we conclude that this signal should persist at these times ( i.e., $t \in [2\times 10^4,7\times 10^4]$ ) as ${L\rightarrow \infty }$. We conjecture that the aforementioned contribution to the domain growth from the annihilation of domains inside domains may be the cause of this superdiffusive behavior with $\alpha _i > 1/2$.

To assure that this behavior is not an artifact from the concurrent spin update caused by the checkerboard decomposition of the system, we repeated our measurements of the domain size $\ell (t)$ with a number of different update algorithms including an efficient *n*-fold way simulation^28^ for system sizes up to $L=1536$ and found agreement within error bars; see Supplementary Discussion II for a comparison of the results from the algorithms and Supplementary Methods I for a discussion of their implementations.

By studying significantly larger system sizes than available in the literature, we thus discover yet another twist in the coarsening story of the three-dimensional Ising model at zero temperature. We find strong evidence for $\alpha _i(t)$ at least pre-asymptotically taking values significantly larger than 1/2 which is in conflict with previous numerical conjectures that $\alpha = 1 / 2$ using smaller systems^23,24^; thus again challenging our understanding of the dynamics in this simple model. (The maximal value obtained for $\alpha _i$ exceeds 1/2 by four [three] times the standard error for $L=2048$ [$L=1536$].) The structure of the domains has been described as sponge-like^20^ or fractal^27^. Anomalous diffusion, including both sub- and superdiffusion, is a well known phenomenon on fractal structures^29^. Hence, we believe that the peculiar structure of domains found in this coarsening problem is both the reason for the early time behavior with $\alpha _i<1/2$ and the late-time stage with $\alpha _i>1/2$. It is nonetheless possible, that we recover $\alpha =1/2$ in the thermodynamic limit, that is in the double limit of $L\rightarrow \infty$ and $t\rightarrow \infty$.

**Figure 4:**
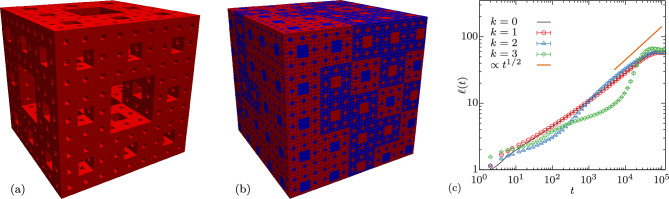
Results for zero-temperature coarsening using an artificial sponge structure as starting configuration. (**a**) Third iteration ($k=3$) Menger sponge of size $27^3$. (**b**) $128^3$ initial Ising configuration consisting of third iteration Menger sponges, red (blue) corresponding to up (down) spins. (**c**) Length scale $\ell (t)$ of a quench to $T=0$ starting from such an artificial configuration using Menger sponges of $k^\text {th}$ iteration and using $L=512$. Error bars correspond to the standard error.

To test our intuition that the sponge-like behavior is responsible for this superdiffusive growth, we carry out one further test. We replace the initial high-temperature random configuration by an artificial sponge structure to probe its effect on the dynamics. As a prototypical sponge structure we use Menger sponges^30^, the three-dimensional generalization of Sierpinski carpets [see Fig. 4a]. The starting configuration [see Fig. 4b] of dimensions $L^3$ is created by repeating sponges of $k\text {th}$ iteration of size $(3^k)^3$. For each sponge we pick uniformly at random whether the Menger structure is represented by up or down spins.

We carry out a zero-temperature quench on these structures in the same manner as before but using the *n*-fold way update^28^ (see Supplementary Methods I C for more detail) instead as this choice avoids potential interference of the Menger sponge structure with the structure of the checkerboard decomposition. From this we obtain the characteristic length scale $\ell (t)$ presented in Fig. 4c. Clearly, the significant differences between $k=0$ (corresponding to our case from before, i.e., a quench from $T=\infty$) and higher-order fractals with $k\ne 0$ become more pronounced the larger *k*. We note two key effects: On the one hand, at early times the dynamics for $k\ne 0$ is much slower than in the $k=0$ case and on the other hand, at later times it becomes much faster than the original dynamics and clearly exceeds a growth governed by $\propto t^{1/2}$. From this we learn that indeed sponge-like structures can cause anomalously slow early dynamics followed by superdiffusive growth at later times which is reminiscent of our observation when quenching the three-dimensional Ising model from infinite to zero temperature.

To conclude, we have simulated zero-temperature coarsening of the three-dimensional Ising model with nearest-neighbour interactions. For this model, the growth exponent of the characteristic length scale is predicted to be 1/2, whereas most simulations previously suggested a smaller exponent $\approx 1/3$. Using a highly efficient GPU implementation, we simulate this process and are able to go to linear system sizes of $L=2048$, i.e., over 8 billion spins. This allows us to monitor late times which previously were not accessible and we discover a previously unknown superdiffusive growth behavior which we attribute to the annihilation of sponge-like structures emerging at early times.

Although we expect 1/2 for the growth exponent in the long-time limit, we cannot fully verify this expectation. This is due to the presence of pre-asymptotic effects at late times even for very large systems. Based on preliminary investigations (not presented here) we are confident that we may get access to the necessary hardware to study even larger systems, i.e., $L = 4096$, in the near future. Additionally, very recent work^31^ reported on the anomalously slow growth prevailing even for quenches to $T>0$ as long as the quench temperature is well below the roughening transition temperature $T_R$. We will investigate in future work whether also the superdiffusion-like behavior is seen at these temperatures.

The idea of making use of GPUs for nonequilibrium investigations using MC simulations is expected to spark investigations of bigger systems also for related spin models. While GPU implementations of MC simulations of spin models have been used and studied in the equilibrium context for several years^32–39^, the potential of application of this approach to nonequilibrium simulations has not been fully realized. One possible explanation for this neglect in nonequilibrium studies is the necessary reliance on checkerboard decomposition to speed up the simulations on the GPU, which some may suspect to introduce artifacts in the dynamics of the simulation. However, with our work we demonstrate that this fear appears to be unfounded and various GPU update methods are indeed suitable for nonequilibrium studies.

## Methods

### Spin updates

In the following we discuss the checkerboard update as an alternative to the random-site-flip update. For details on further alternative update methods, we refer to Supplementary Methods I.

#### Random-site-flip update

The random-site-flip (rsf) update is the most straightforward method to perform MC simulations and to study coarsening in the Ising model. In each MC step one chooses a site *i* at random and proposes to flip the spin $\sigma _i \in \{-1, +1\}$. Based on the change in energy $\Delta E$ attributed to the proposed move, in general for non-zero temperature *T* it is accepted with the Glauber acceptance probability^40^

5 $$\begin{aligned} p_\text {acc}(\Delta E,T) = \frac{1}{1+e^{\Delta E / k_B T}}, \end{aligned}$$

where the Boltzmann constant $k_B$ usually is set to unity to fix units. In the limit $T\rightarrow 0$ this simplifies to

6 $$\begin{aligned} p_\text {acc}(\Delta E) = {\left\{ \begin{array}{ll} 0, &{}\, \Delta E > 0 \\ \frac{1}{2}, &{}\, \Delta E = 0 \\ 1, &{}\, \Delta E < 0 \end{array}\right. }, \end{aligned}$$

which is the acceptance probability we use throughout. $N=L^3$ such MC steps are referred to as one MC sweep (MCS), where *L* is the linear lattice size.

Clearly, this approach is rather inefficient as *(i)* a significant amount of computing resources is wasted on proposing moves with $\Delta E > 0$ (Ref.^20^), which always are rejected, and *(ii)* because it is inherently sequential making it hard to parallelize the algorithm.

#### Checkerboard update

The key idea of many domain-decomposition spin update algorithms such as the checkerboard update is that with local, i.e., short-range, interactions only, the lattice can be decomposed into sub-lattices such that spins of the same group do not interact with one another.

**Figure 5:**
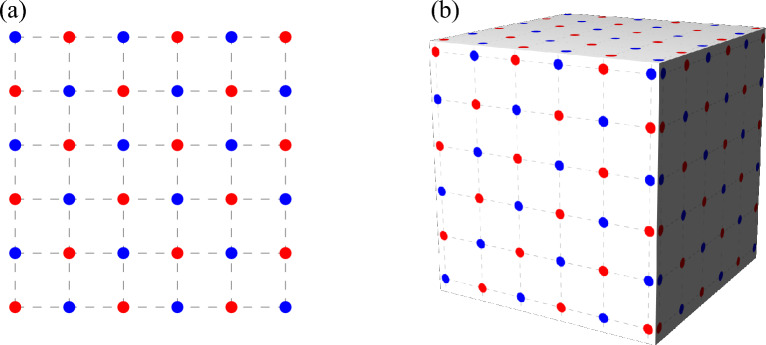
Checkerboard decomposition. All red (blue) sites can be updated simultaneously as they only depend on blue (red) sites. (**a**) Checkerboard decomposition in two spatial dimensions. (**b**) Generalization to three spatial dimensions.

In the case of the two-dimensional square lattice with only nearest-neighbor interactions one of the simplest such decompositions looks like a checkerboard (see Fig. 5a), hence the name of the method. One MC sweep then consists of *(i)* updating all red spins concurrently with *N*/2 parallel threads, followed by *(ii)* updating all blue spins concurrently with *N*/2 parallel threads. Equivalently, one may choose to update all blue spins first (see Supplementary Methods I B for more detail). Each proposed spin flip is accepted with the same probability as before [see Eq. (6)] and the only difference as compared to the rsf update is the order in which updates are proposed. The generalization to $d=3$ is conceptually straightforward, see Fig. 5b.

This update scheme is particularly suited for an implementation on graphics processing units (GPUs). GPUs have several thousand threads which can be used to update the independent spins in parallel. Our implementation in CUDA for this update scheme is based on the code from Ref. ^25^ although heavily adapted as the authors optimized their code for $>1000$ simultaneous simulations of small systems on a single GPU. In contrast, we simulate a single large system per GPU. Additionally, Ref. ^25^ considers the two-dimensional Ising model. Hence, the respective parts of the code have been modified accordingly.

### Calculating the correlation function

Naïve calculation of the correlation function defined in Eq. (2), i.e., $C(r,t) = \langle s_i s_j \rangle - \langle s_i\rangle \langle s_j\rangle ,$ involves a double summation over all spins requiring ${\mathscr {O}}(N^2)$ time. Using a Fast Fourier Transform (FFT) allows the calculation of $\langle s_i s_{i+k} \rangle$ in ${\mathscr {O}}(N \log N)$, i.e.,

7 $$\begin{aligned} \overline{s_i s_{i+k}} = [{\mathscr {F}}^{-1}(|{\mathscr {F}} s_i|^2)]_k, \end{aligned}$$

where ${\mathscr {F}}$ is the three-dimensional discrete Fourier transformation operator and the overline stands for an average over *i*, exploiting the translational invariance. *C*(*r*, *t*) shown in the main text is then obtained by radially averaging over the three-dimensional correlation matrix.

In standard FFT routines two double values are used per (spin) site both for input and output, requiring thus $4\times 8$ bytes per spin. For $L=2048$ this amounts to $4\times 8 \times 2048^3= 2^{38}$ bytes $= 256$ GB RAM necessary to carry out the FFT which on modern CPU computing nodes is possible but still quite restrictive as it limits the number of simulations which can be run in parallel on the same node. We use the FFTW library^41^ which allows for in-place calculation such that the memory footprint is cut in half.

Further, spin variables only take the values $-1$ and $+1$, and $|{\mathscr {F}} s_i|^2$ in Eq. (7) only real values. Hence, the real-data discrete Fourier transform (DFT) routine can be used for both transforms which reduces the used memory by another factor of two by using that the resulting DFTs are Hermitian. This allows the input to be stored in *N* double values and the output to be stored in *N*/2 complex (= two doubles) values. When using in-place real-data DFT, this brings the memory footprint down to eight bytes per site such that for the FFT of one $2048^3$ lattice only 64 GB RAM are necessary which are readily available on our compute nodes. However, already for the next bigger system size, i.e., $L=4096$, we can no longer compute the FFT in the same manner as $64 \times 8 = 512 \text { GB}$ RAM are not available to us.

Additionally, FFTW supports multi-threading such that we can speed up the calculation by a factor of about 10 compared to the sequential algorithm.

### Supplementary Information

Supplementary Information.

## Data Availability

The data that support the findings of this study are available from the corresponding author upon reasonable request.
