# Supplementary material for: Superdiffusion-like behavior in zero-temperature coarsening of the d = 3 Ising model
Source: Sci Rep. 2023 Aug 15;13:13270. doi: 10.1038/s41598-023-39328-7 (PMC10427613; doi:10.1038/s41598-023-39328-7)
Supplement: Supplementary file 1 — Supplementary Information. [file 41598_2023_39328_MOESM1_ESM.pdf]

Supplementary Material: Superdiffusion-like behavior in zero-temperature coarsening of the $d = 3$ Ising model

Denis Gessert,[1,2,*] Henrik Christiansen,[1,†] and Wolfhard Janke[1,‡]

[1] *Institut für Theoretische Physik, Universität Leipzig, IPF 231101, 04081 Leipzig, Germany*
[2] *Centre for Fluid and Complex Systems, Coventry University, Coventry CV1 5FB, United Kingdom*

(Dated: April 28, 2023)

SUPPLEMENTARY METHODS: ALTERNATIVE SPIN UPDATES

We study coarsening, a nonequilibrium process, in the three-dimensional Ising model with non-conserved order parameter by means of Monte Carlo (MC) simulations. As it is not uniquely specified how to perform spin updates, there are several ways to realize the underlying Markov chain. In the case of nonequilibrium investigations clearly one cannot take advantage of non-local updates such as the Wolff cluster algorithm. Nonetheless, for spin models and local updates, the way in which the single-spin updates are proposed is not stipulated. Instead, one has a choice to adapt this, e.g., to speed up simulations. In the following we present different approaches to implement spin updates alternative to the random-site-flip update and checkerboard update which are discussed in the main text. At the end of this section we will check numerically that all the considered approaches produce dynamics that are compatible (within error bars) up to a rescaling of time by a constant factor.

Double-checkerboard update

Weigel [1] introduced a slightly more involved domain-decomposition scheme which aims at being more efficient on GPUs than the standard checkerboard approach. On GPUs, threads are organized in so-called thread blocks that can synchronize locally whereas threads from different blocks essentially cannot communicate within one execution of the GPU kernel. For the performance of the algorithm it is beneficial to use the much faster shared memory of these blocks rather than the global memory available to all blocks. However, the shared memory is too small to even fit systems of moderate size. Thus, the idea of the double-checkerboard decomposition is to divide the system up into a coarse checkerboard (red and blue blocks in Fig. 1) of small sub-systems which still can fit in the shared memory. These can then be updated by a thread block using the standard checkerboard (lighter and darker sites in Fig. 1).

One MC sweep using the double-checkerboard scheme based on the decomposition depicted in Fig. 1 then consists of the following steps:

1. Start GPU kernel on red sublattice.

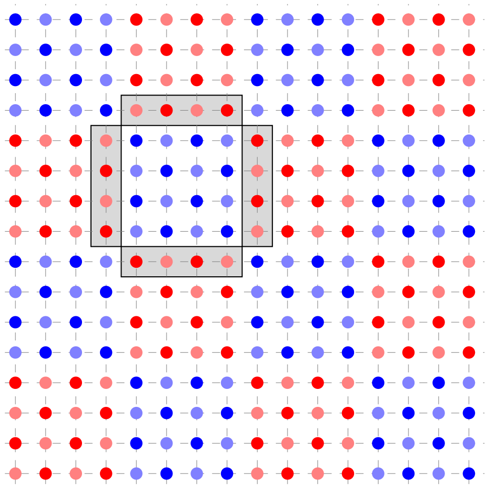

FIG. 1. Example double-checkerboard decomposition in $d = 2$.

2. Threads load spins into shared memory.
3. Glauber update on darker spins.
4. Barrier synchronization within thread blocks.
5. Glauber update on lighter spins.
6. Barrier synchronization within thread blocks.
7. Writing changes to global memory.
8. Start GPU kernel on blue sublattice and repeat steps 2 to 7.

Our implementation is mostly based on the publicly available code of Ref. [1]. Similar to checkerboard discussed in the main text, the generalization to three spatial dimensions was performed.

Variants of the single- and double-checkerboard update

A priori it is not immediately clear that any of the described domain decomposition updates should exhibit the same kind of coarsening dynamics as the rsf update. In fact, there are at least three problems we are aware of regarding this type of update: *i)* In contrast to rsf, no spin is proposed to be flipped more than once within

a sweep, *ii)* the violation of detailed balance, and *iii)* extreme non-ergodicity at infinite temperature.

Regarding problem *i)*, using the rsf update the chance for every spin in a system of N spins to be proposed to be flipped exactly once during a sweep is $N!/N^N$ which approaches $\sqrt{2\pi N}e^{-N}$ for large N. For a 2^3 system this is approximately 0.2% and already for 3^3 around 2×10^{-11}. Thus, for the system sizes we study, it is nearly impossible not to have a repetition. Through generalization of the birthday problem [2] we expect a repetition after $\mathcal{O}(\sqrt{N})$ proposals, that is $\mathcal{O}(\sqrt{N})$ repetitions per sweep.

Problem *ii)* can in principle be circumvented. Potter and Swendsen [3] previously reported a violation of detailed balance on a broad set of update algorithms such as the sequential (typewriter) update and the checkerboard update. While in the context of nonequilibrium detailed balance is not a really meaningful concept, it led us to wonder whether the checkerboard dynamics that violates detailed balance might be different from the rsf dynamics satisfying detailed balance. Reference [3] further introduces variants of the algorithms which they prove do satisfy detailed balance. In the standard checkerboard algorithm the two sublattices are always updated in the same order, which they refer to as the *01 cycle*. They claim that through a simple change, namely choosing at random which sublattice to update first, detailed balance can be reestablished. Note that still every spin is proposed to flip exactly once per sweep. This is referred to as *mixed cycle*. For the double-checkerboard the order of the coarse checkerboards (red or blue first) is chosen at random once per sweep and the order of the fine checkerboard (lighter or darker sites first) is chosen at random and independently for each (red or blue) block and once per sweep. Reference [3] proves that detailed balance is reestablished in this way in a general setting that entails many domain decomposition schemes including the double-checkerboard although not explicitly mentioned.

Lastly, at infinite temperature and using the Metropolis acceptance criterion $p_{\mathrm{acc}}(\Delta E, T) = \min\{1, e^{-\Delta E/k_B T}\}$ all spin flip proposals are always accepted leading to problem *iii)*. Consequentially, when every spin is proposed to flip once per sweep then one sweep consists of inverting the lattice. Hence, the system just switches between two states. Technically, this problem does only occur at infinite temperature but nonetheless causes large autocorrelation times at large finite temperatures. Reference [4] comments on this issue but using the typewriter update instead of the checkerboard update. There spins are also proposed once per sweep (but in lexicographical order), and hence suffering the same fate as the checkerboard update at infinite temperature.

n-fold way update

At low temperatures, the problem of high rejection rates is well known (see, e.g., ch. 3.4.2 in Ref. [5]). Particularly in the case of $T = 0$, many proposed spin flips will be rejected with 100% certainty.

Already in the 1970s this problem was tackled by Bortz *et al.* [6] with the introduction of the rejection-free n-fold way update algorithm. They recognized that instead of selecting each site with the same probability (as is the case in rsf) the chance of a site to be selected can be weighted by the acceptance probability of a spin flip which is then always accepted. This can be implemented very efficiently for systems with discrete spin variables and local interactions only by organizing spins in n spin classes of equal acceptance probability. In our case $n = 2 \times (6 + 1)$, as each site can take two values and the sum of its six nearest neighbor's spin values can take seven different values. To have a unit of time in MCS and not the number of successful flips one increments time after every flip by a geometrically distributed random variable with mean $N/\sum_i p_\beta([\Delta E]_i)$ where $p_\beta(\Delta E)$ being the usual acceptance probability if rsf were used [7].

One way of improving the performance of n-fold way is to use continuous time steps instead of geometrically distributed ones, that is to increment time by the expected time. The difference between summing over many geometrically distributed variables and summing over their means is negligible on the time scales considered here which motivates this simplification.

The n-fold way update has been used to study coarsening before, see for example Refs. [6, 8, 9]. In the case of the $T = 0$ coarsening in $d = 3$, Olejarz *et al.* [10] previously have used a very similar approach. Instead of complete rejection-free updates they pick at random from the set of allowed spin flips, i.e., they randomly propose to flip a spin from the set $\{\sigma_i | [\Delta E]_i \leq 0\}$. If $\Delta E < 0$ the move is immediately accepted and otherwise if $\Delta E = 0$, the move is accepted with 50% probability.

SUPPLEMENTARY DISCUSSION: NUMERICAL COMPARISON OF ALTERNATIVE SPIN UPDATES

In the following we present data obtained through the various different spin update schemes outlined above, where for the double-checkerboard we have tested two different cycles one of which preserves detailed balance. Every dataset is averaged over at least 40 independent realizations. The autocorrelation time is sensitive to the type of spin update employed. Similarly, the time scale in coarsening is affected by the chosen update. We used the dynamics from the rsf update as a base and then rescaled all other observation times by a factor specific to the used method resulting in a rescaled time scale t,

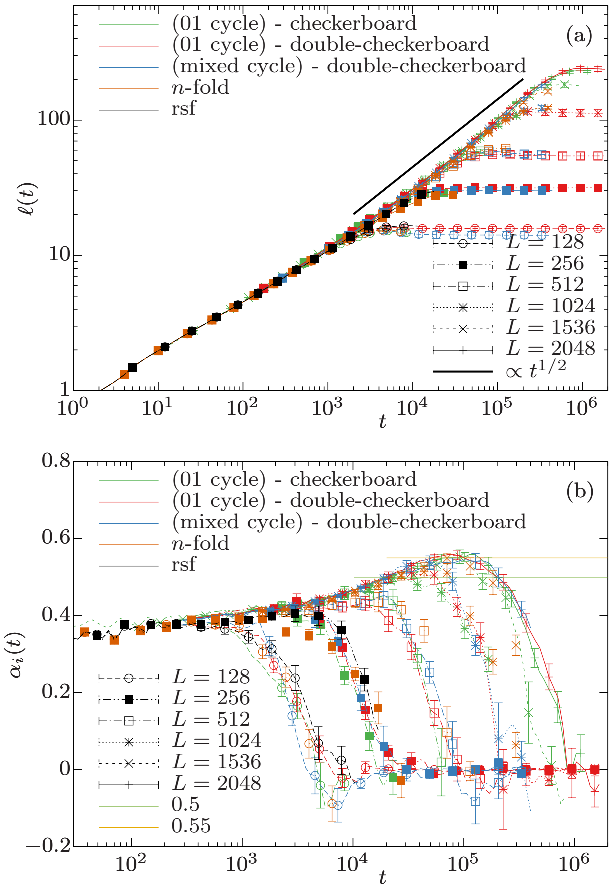

FIG. 2. (a) Length scale $\ell(t)$ and (b) instantaneous growth exponent $\alpha_i(t)$ for various spin update methods obtained for quenches to $T = 0$. In both plots the color of the line encodes the used method and the line type and symbol the system size. Note that for all methods (except rsf and n-fold) time is rescaled by a constant factor for better comparison (cf. Table I). For sake of readability, only every fifth data point is shown and lines are a guide to the eye connecting both shown and not shown points by straight lines. Error bars correspond to the standard error.

see Table I for the factors.

Figure 2(a) shows the domain size and (b) the instantaneous growth exponent as a function of (rescaled) time t. It can be seen that within error bars all the methods produce compatible results.

In particular all simulations show a growth exponent as large as $\simeq 0.55$ at late times. Using the n-fold way update, only linear system sizes of up to 1536 were accessible to us, which also display the anomalous growth exponent larger than 0.5. Hence, we can rule out that the larger exponent is a mere effect from the parallelized GPU update algorithms. Furthermore, this shows that the observation is in fact relevant in the thermodynamic limit and not just a finite-size effect. To check our n-fold way update implementation for correctness we used the rsf with which systems of linear size up to about 256

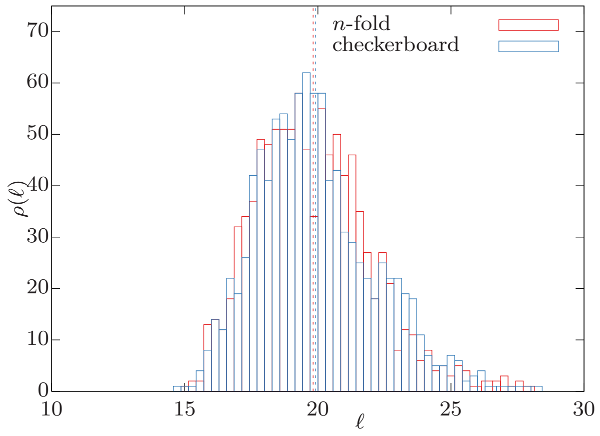

FIG. 3. Empirically determined domain-size histogram for $L = 256$ at rescaled time $t = 4000$ MCS using the n-fold way update and the (01-cycle) checkerboard decomposition. Data was obtained through 1000 independent runs for each method. Dashed vertical lines show the mean value of the two histograms, that is 19.8 for n-fold way and 19.9 for the checkerboard update.

are (easily) accessible. Note that due to the significant computing time required to simulate systems as large as $N = 1536^3$ on CPUs we refrained from simulating past the onset of finite-size effects which is why the $L = 1536$ line ends earlier than the other data sets displayed.

Finally, we run 1000 simulations with system size $L = 256$ once using the (01-cycle) checkerboard update and once using the n-fold way update. The first method is the one we used to obtain the data presented in the main text and the latter is known to obey the same dynamics as rsf Glauber dynamics. In Fig. 3 we present a histogram of the measured domain sizes at a rescaled time 4000 MCS (well before the onset of finite-size effects). The histograms appear to be in good agreement.

TABLE I. Factors by which simulation times in Fig. 2 were multiplied to compare them. A higher number implies quicker dynamics.

Method	Factor
(01 cycle) – checkerboard	1.75
(01 cycle) – double-checkerboard	1.75
(mixed cycle) – double-checkerboard	1.45
n-fold	1
rsf	1

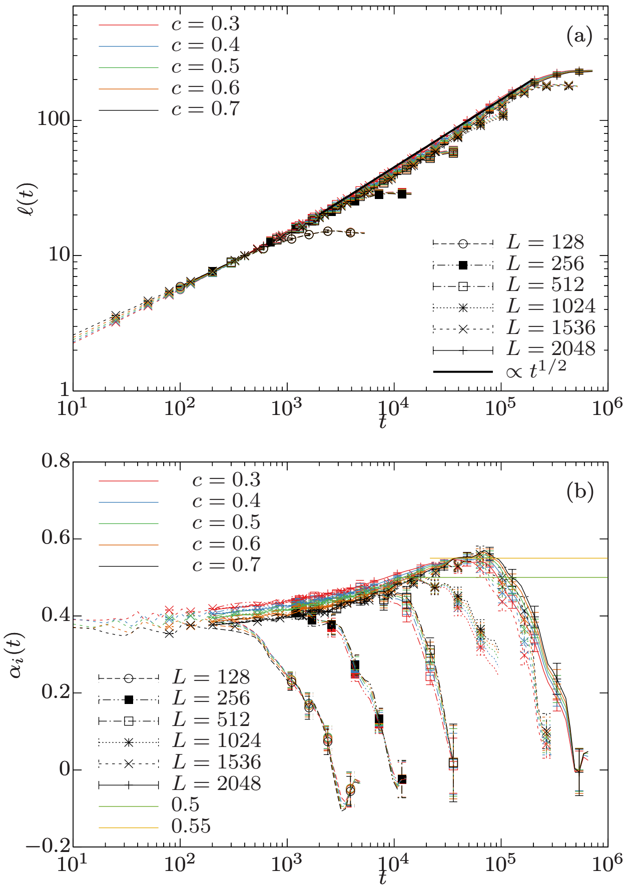

FIG. 4. (a) Length scale $\ell(t)$ and (b) instantaneous growth exponent $\alpha_i(t)$ for various choices of the correlation-function intercept c. In both plots the color of the line encodes the used intercept and the line type and symbol the system size. Note that for all intercepts, the length scale is rescaled by a constant factor for better comparison (see Table II). For sake of readability, only every fifth data point is shown and lines are a guide to the eye connecting both shown and not shown points by straight lines. Error bars correspond to the standard error.

SUPPLEMENTARY DISCUSSION: COMPARISON OF DIFFERENT INTERCEPT VALUES AND EFFECT ON THE CALCULATED DOMAIN SIZE

In the following we repeat the measurement of the characteristic length scale $\ell(t)$ and vary the chosen intercept c with $C(r,t)$. $\ell(t)$ is then the distance r at which $C(r,t)$ intercepts c. We use the (01-cycle) checkerboard update (same as in the main text).

Figure 4 shows the different results for (a) $\ell(t)$ and (b) $\alpha_i(t)$. The characteristic length scales $\ell(t)$ for the various intercepts differ only by a constant factor and exhibit approximately the same $\alpha_i(t)$. To better show this, $\ell(t)$ is rescaled by this constant (independent of time and system size) for each intercept. Note, that this constant does not affect the obtained value for α_i. The length scales obtained in this way are compatible with each other, thus confirming that the exact choice of c is of little importance. Most importantly, note that all datasets for $L > 1\,024$ and any intercept c show $\alpha_i > 0.5$ at late times, thus confirming our observations in the main text. The times t in Fig. 4 correspond to the not rescaled times. Hence, the data can readily be compared to Fig. 3 in the main text but times differ by a factor of 1.75 to the ones in Fig. 2 in this supplement. The factors by which the estimates for ℓ from each intercept were multiplied such that the data sets collapse are compiled in Table II. Further, Table II shows the length scale ℓ^* at which $\ell(t)$ would saturate *without rescaling.*

THREE-DIMENSIONAL VISUALIZATION

In Fig. 5 we show snapshots for different times in the evolution of the spin configuration from a single n-fold way simulation run with $L = 1536$. In this case we chose the n-fold way update instead of the checkerboard in an attempt to check whether any (obvious) structural differences can be seen. We make use of the software Visual Molecular Dynamics (VMD) [11] to illustrate the three-dimensional spin configurations. In the cubic representation (left column) a spin is represented either by a cube or the lack thereof. To make the plotting tractable, we choose to represent the minority direction as a cube (such that we always have to display less than $N/2$ cubes). This representation directly shows the shape of typically encountered domains, but most of the inside of the lattice is hidden. For a better illustration of the inside of the lattice, in the interface representation (center column) we only draw lines connecting the spins at the domain boundaries. The color of the interfaces represents the distance from the center of the lattice (this is artificial, since periodic boundary conditions apply), where we employ a hot (red, center) to cold (blue, border) color palette. This allows for a deeper look inside the lattice and highlights the nature of the domain boundaries, in particular the sponge-like structure. Finally, we show two-dimensional

TABLE II. **Rescaling factors and saturation lengths for different intercepts.** The middle colum shows the factors by which the length scales in Fig. 4 were multiplied to compare them. The right column shows the length ℓ^* at which the not rescaled $\ell(t)$ estimate saturates.

**Intercept** c	**Factor**	**Saturation Length** ℓ^*
0.3	0.61	$0.19\,L$
0.4	0.77	$0.15\,L$
0.5	1	$0.115\,L$
0.6	1.34	$0.086\,L$
0.7	1.90	$0.061\,L$

cuts through the lattice (right column).

* denis.gessert@itp.uni-leipzig.de
† henrik.christiansen@itp.uni-leipzig.de; Present address: NEC Laboratories Europe GmbH, Kurfürsten-Anlage 36, 69115 Heidelberg, Germany.
‡ wolfhard.janke@itp.uni-leipzig.de

[1] Martin Weigel, "Simulating spin models on GPU," Comput. Phys. Comm. **182**, 1833 (2011); the source code can be found at the author's personal homepage on `http://www.martin-weigel.org/research/gpu-computing`.

[2] Frank H. Mathis, "A generalized birthday problem," SIAM Review **33**, 265–270 (1991).

[3] Christopher C. J. Potter and Robert H. Swendsen, "Guaranteeing total balance in Metropolis algorithm Monte Carlo simulations," Physica A **392**, 6288–6299 (2013).

[4] Martin Weigel, Lev Barash, Lev Shchur, and Wolfhard Janke, "Understanding population annealing Monte Carlo simulations," Phys. Rev. E **103**, 053301 (2021), arXiv:2102.06611 [cond-mat.stat-mech].

[5] S. Puri, "Kinetics of phase transitions," in *Kinetics of Phase Transitions*, edited by S. Puri and V. Wadhawan (CRC Press, Boca Raton, 2009).

[6] A. B. Bortz, M. H. Kalos, and J. L. Lebowitz, "A new algorithm for Monte Carlo simulation of Ising spin systems," J. Comput. Phys. **17**, 10 (1975).

[7] One can easily convince oneself, that if the success probability of a single rsf spin proposal is p_{succ}, then the number of tries necessary to make a flip (irrespective of what site is flipped) follows a geometric distribution with mean $1/p_{\mathrm{succ}}$.

[8] Erik Schwartz Sørensen, Hans C. Fogedby, and Ole G. Mouritsen, "Crossover from nonequilibrium fractal growth to equilibrium compact growth," Phys. Rev. Lett. **61**, 2770–2773 (1988).

[9] T. Blanchard, F. Corberi, L. F. Cugliandolo, and M. Picco, "How soon after a zero-temperature quench is the fate of the Ising model sealed?" Europhys. Lett. **106**, 66001 (2014).

[10] J Olejarz, P L Krapivsky, and S Redner, "Zero-temperature relaxation of three-dimensional Ising ferromagnets," Phys. Rev. E **83**, 051104 (2011).

[11] William Humphrey, Andrew Dalke, and Klaus Schulten, "VMD: Visual molecular dynamics," J. Mol. Graph. **14**, 33–38 (1996).

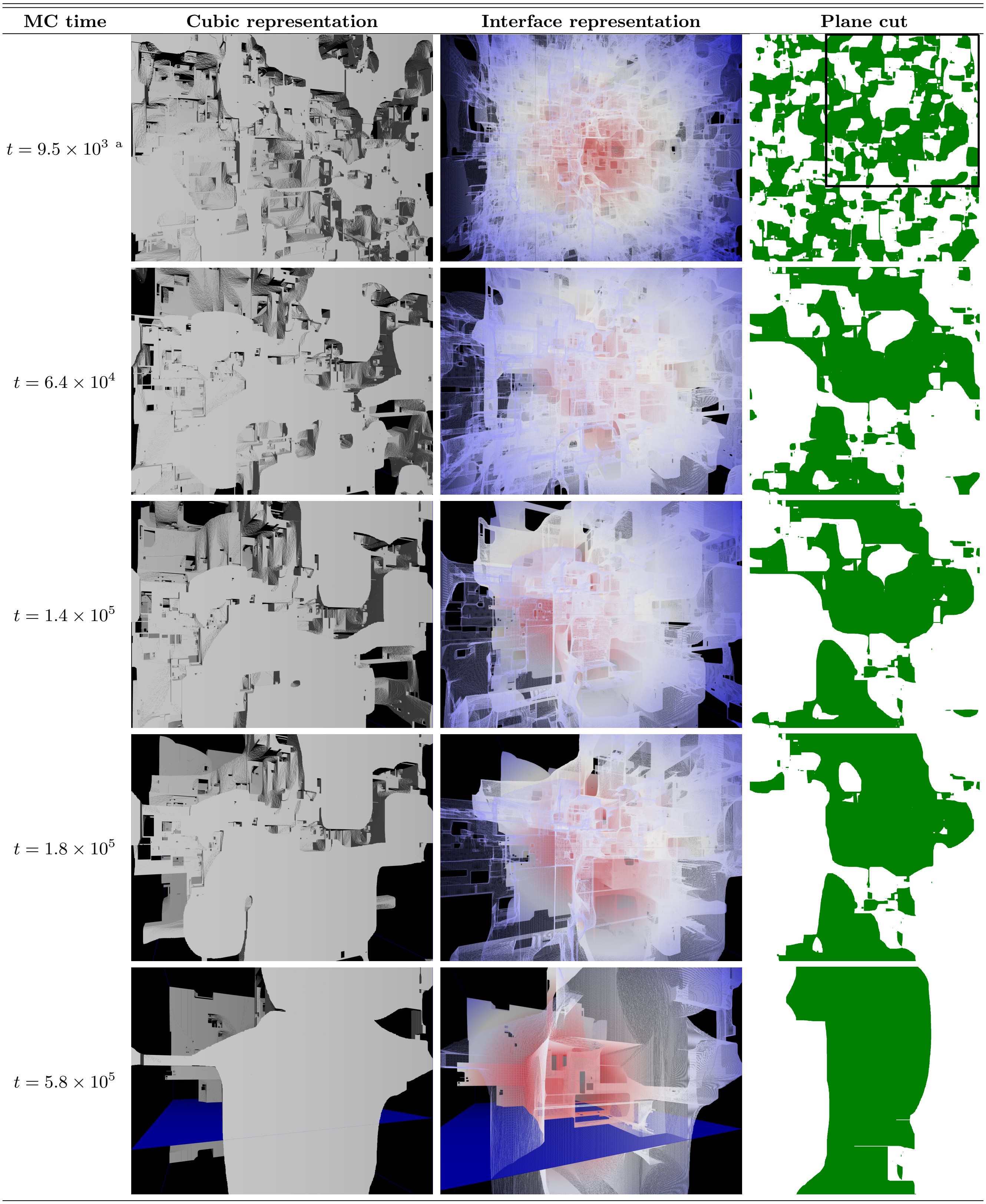

[a] Instead of the full 1536^3 snapshot we show a 1024^3 subsection for the earliest time. This is on the one hand because otherwise the small features are hard to see and on the other hand because VMD appears to fail to handle the large number of "atoms" present in these early-time configurations. The cross-section shown in the third column has length $L = 1536$ and the 1024 square highlights the region visible in the 3D representations.

FIG. 5. Visualizations of snapshots obtained during a quench using $L = 1536$. The plane cut is a horizontal slice from the three-dimensional structure, which for $t = 5.8 \times 10^5$ is highlighted in the three-dimensional representations as the blue planes.
